# Medical Examinations in London

**Published:** 1853-06

**Authors:** 


*Medical Examinations in London.*—In a former No. of this Jourual, we copied an article from a London correspondent of the Dublin Medical Press, describing the condition of the medical profession in the English metropolis. We now give a quotation from the same source, in which the medical examinations in London are noticed. We beg leave to commend the following extract to those who are fond of disparaging the course pursued in this country in determining the qualifications of candidates for medical degrees, by a comparison with the European customs:

"Examinations, particularly at the College, are notoriously a sham. Nine men, or nineteen, (so goes the story,) at present go up from Bartholomew's, and sporting bets are laid against Mr. Guthrie that the entire batch passes. One celebrated grinder risks any sum that he will get a man who knows absolutely nothing, and pass him in three months. All oldish men pass as a matter of course; the Board, with paternal solicitude, say, 'If this fellow does not pass and pay *us* his twenty guineas, he is sure to waste it, and practice as a quack.' Such the moral hold of Examining Boards and Colleges on the mass of English students. King's College men say they are 'plucked,' because one of their men puzzled the College itself about some 'pin-hole' question of the bones of the face; while the St. George's and Guy's men count their chances on the Examination Board with the same certainty that one counts votes on a division in the House of Commons. Of course, in the select society of all grinders and grinding students, there are stories of this kind; but in London it pervades everything, in the schools and museums. We were shown a common sailor who passed in one of the last batches at the College: this would be impossible in Dublin. In all this, however, we would wish to be understood as attacking *a system*, not individual members of the Board, whom we hold in highest esteem."

